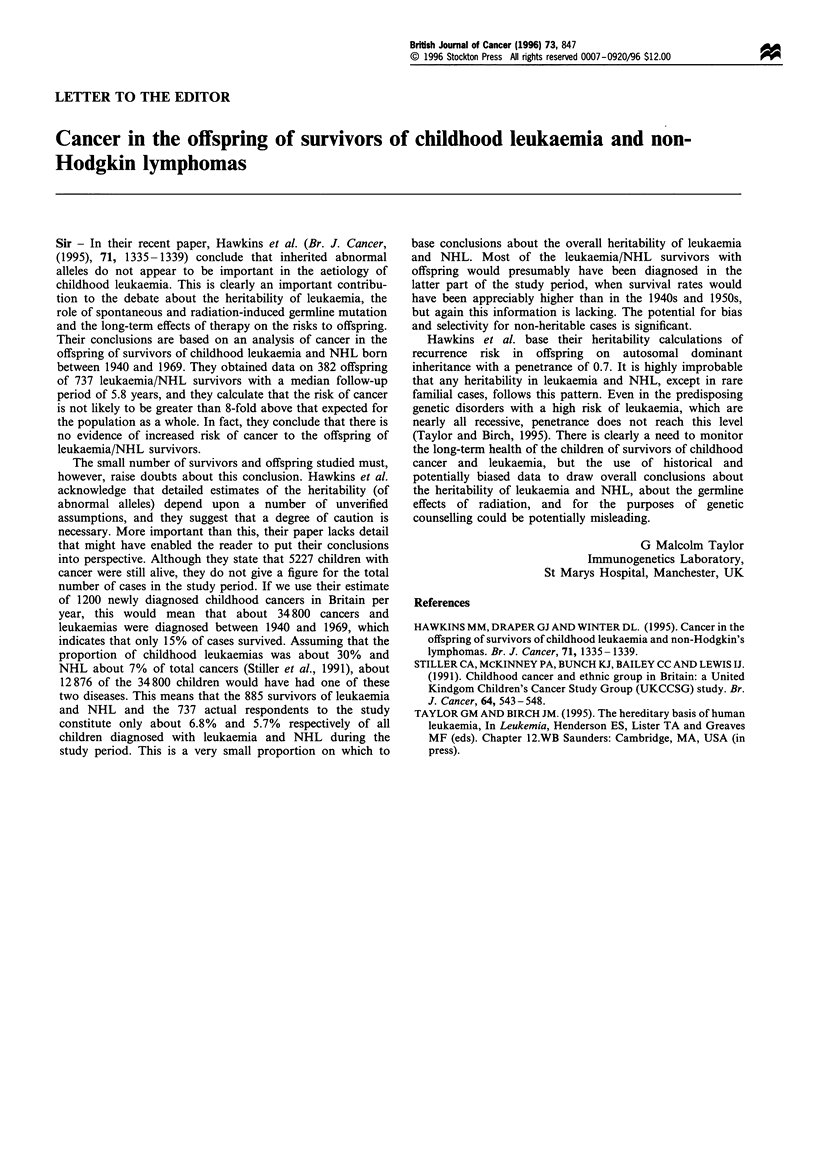# Cancer in the offspring of survivors of childhood leukaemia and non-Hodgkin lymphomas.

**DOI:** 10.1038/bjc.1996.149

**Published:** 1996-03

**Authors:** G. M. Taylor


© 1996 Stockton Press All rights reserved 0007-0920/96 $12.00

LETTER TO THE EDITOR

### Cancer in the offspring of survivors of childhood leukaemia and non-Hodgkin lymphomas

**Sir** – In their recent paper, Hawkins *et al.* (*Br. J. Cancer*, (1995), **71,** 1335–1339) conclude that inherited abnormal alleles do not appear to be important in the aetiology of childhood leukaemia. This is clearly an important contribution to the debate about the heritability of leukaemia, the role of spontaneous and radiation-induced germline mutation and the long-term effects of therapy on the risks to offspring. Their conclusions are based on an analysis of cancer in the offspring of survivors of childhood leukaemia and NHL born between 1940 and 1969. They obtained data on 382 offspring of 737 leukaemia/NHL survivors with a median follow-up period of 5.8 years, and they calculate that the risk of cancer is not likely to be greater than 8-fold above that expected for the population as a whole. In fact, they conclude that there is no evidence of increased risk of cancer to the offspring of leukaemia/NHL survivors.

The small number of survivors and offspring studied must, however, raise doubts about this conclusion. Hawkins *et al.* acknowledge that detailed estimates of the heritability (of abnormal alleles) depend upon a number of unverified assumptions, and they suggest that a degree of caution is necessary. More important than this, their paper lacks detail that might have enabled the reader to put their conclusions into perspective. Although they state that 5227 children with cancer were still alive, they do not give a figure for the total number of cases in the study period. If we use their estimate of 1200 newly diagnosed childhood cancers in Britain per year, this would mean that about 34 800 cancers and leukaemias were diagnosed between 1940 and 1969, which indicates that only 15% of cases survived. Assuming that the proportion of childhood leukaemias was about 30% and NHL about 7% of total cancers (Stiller *et al.*, 1991), about 12 876 of the 34 800 children would have had one of these two diseases. This means that the 885 survivors of leukaemia and NHL and the 737 actual respondents to the study constitute only about 6.8% and 5.7% respectively of all children diagnosed with leukaemia and NHL during the study period. This is a very small proportion on which to base conclusions about the overall heritability of leukaemia and NHL. Most of the leukaemia/NHL survivors with offspring would presumably have been diagnosed in the latter part of the study period, when survival rates would have been appreciably higher than in the 1940s and 1950s, but again this information is lacking. The potential for bias and selectivity for non-heritable cases is significant.

Hawkins *et al.* base their heritability calculations of recurrence risk in offspring on autosomal dominant inheritance with a penetrance of 0.7. It is highly improbable that any heritability in leukaemia and NHL, except in rare familial cases, follows this pattern. Even in the predisposing genetic disorders with a high risk of leukaemia, which are nearly all recessive, penetrance does not reach this level (Taylor and Birch, 1995). There is clearly a need to monitor the long-term health of the children of survivors of childhood cancer and leukaemia, but the use of historical and potentially biased data to draw overall conclusions about the heritability of leukaemia and NHL, about the germline effects of radiation, and for the purposes of genetic counselling could be potentially misleading.

G Malcolm Taylor
Immunogenetics Laboratory,
St Marys Hospital, Manchester, UK

### References

HAWKINS MM, DRAPER GJ AND WINTER DL. (1995). Cancer in the offspring of survivors of childhood leukaemia and non-Hodgkin's lymphomas. *Br. J. Cancer*, **71,** 1335–1339.

STILLER CA, McKINNEY PA, BUNCH KJ, BAILEY CC AND LEWIS IJ. (1991). Childhood cancer and ethnic group in Britain: a United Kindgom Children's Cancer Study Group (UKCCSG) study. *Br. J. Cancer*, **64,** 543–548.

TAYLOR GM AND BIRCH JM. (1995). The hereditary basis of human leukaemia, In *Leukemia*, Henderson ES, Lister TA and Greaves MF (eds). Chapter 12.WB Saunders: Cambridge, MA, USA (in press).